# Analysis of the full-length genome of a novel strain of the H7N9 avian influenza virus

**DOI:** 10.3892/etm.2014.1590

**Published:** 2014-02-28

**Authors:** XINHUA OU, FAMING CHEN, RUSHENG ZHANG, JINGFANG CHEN, RUCHUN LIU, BIANCHENG SUN

**Affiliations:** Changsha Center for Disease Control and Prevention, Changsha, Hunan 410001, P.R. China

**Keywords:** influenza virus type A, H7N9 strain, genome, sequence analysis

## Abstract

The aim of the present study was to analyze the evolution and variation of a novel strain of the avian influenza virus. The virus-positive specimens [A/Changsha/2/2013 (H7N9)] from a patient infected with the novel avian influenza A (H7N9) virus was amplified by reverse transcription-PCR and the full genome was sequenced. The sequencing results were submitted to GenBank and then analyzed by phylogenetic tree analysis using BioEdit and Mega5 software. The phylogenetic tree of the hemagglutinin (HA) and neuraminidase genes revealed that A/Changsha/2/2013 (H7N9) and all the new H7N9 viruses in 2013 were in a large cluster, and their nucleotide evolutionary distances were closely associated. Phylogenetic tree analyses of the nucleoprotein and nonstructural genes demonstrated two main branches. One branch contained novel H7N9 viruses isolated from avian, human and environmental sources in different regions. The other branch contained three novel H7N9 virus strains isolated from environmental sources in Shanghai. All the phylogenetic trees of the matrix protein, polymerase acidic, polymerase basic protein 1 and polymerase basic protein 2 genes also showed two branches, with each branch including the novel H7N9 virus strains isolated from avian, human and environmental sources in different regions. Molecular characterization demonstrated that 52 novel H7N9 viruses sequenced to date contain the G228S and G186V mutations in the receptor binding site of the HA protein. The full-genome sequences of A/Changsha/2/2013 and analyses of its molecular characteristics suggest that the A/Changsha/2/2013 H7N9 virus strain has molecular characteristics that may facilitate adaptation of the virus to mammalian hosts and may even bind to human receptors.

## Introduction

The avian influenza virus is a member of the *Orthomyxoviridae* family of the *Influenzavirus A* genus. The subtypes capable of infecting humans are H5N1, H7N2, H7N3, H7N7 and H9N2. The majority of avian influenza viruses lead to mild symptoms in infected humans, which mainly include conjunctivitis or upper respiratory tract infection, with the exception of H5N1 infections, which have a mortality rate >50% (1). A new reassortant viral subtype named H7N9 was initially isolated from a patient with a severe lower respiratory tract infection (2). Internal genes of this virus originate from the H9N2 avian influenza virus (2). Patients who are infected with the H7N9 virus usually present flu-like symptoms possibly accompanied by a headache, muscle aches and general malaise. For patients with severe infections, the progression of the disease is rapid. The symptoms include severe pneumonia, hyperthermia (which is mostly sustained at 39°C or above) and difficulty in breathing, and may also be accompanied by hemoptysis. The symptoms may also progress rapidly to acute respiratory distress syndrome and even lead to mortality (3).

As of July 31, 2013, a total of 133 human cases (including 44 cases of mortality) infected with the H7N9 virus had been reported in 41 cities among 11 provinces in mainland China (4). At present, cases occur sporadically. It has been demonstrated that the H7N9 avian flu virus can be transmitted to humans by avians, but with limited human-to-human transmission activity (5). Due to the lack of vaccines and the persistence of the H7N9 virus in chickens (6), there remains a risk of human infection with the H7N9 virus. On April 27, 2013, the H7N9 virus [A/Changsha/2/2013 (H7N9)] was detected from the tracheal aspirates of a patient with severe pneumonia in Changsha, China. In the present study, the whole genome of the A/Changsha/2/2013 virus was sequenced, and the evolution and molecular characteristics of the virus were analyzed.

## Materials and methods

### Patient

A 54-year-old male patient developed a fever and sore throat on April 15, 2013. The diagnostic results indicated that the patient was infected with the H7N9 avian influenza virus, and infection with the H7N9 virus was subsequently confirmed. The patient eventually succumbed to severe disease. This study was approved by the ethics review board of Changsha Center for Disease Control and Prevention (Changsha, China). Prior written and informed consent were obtained from the patient’s family.

### Reverse transcription (RT)-PCR

The A/Changsha/2/2013 (H7N9)-containing tracheal aspirate samples were inactivated at 65°C for 30 min and then used for the viral genome RNA extraction using an RNeasy^®^ Mini kit (Qiagen Inc., Hilden, Germany). Eight gene fragments [polymerase basic protein 2 (PB2), PB1, polymerase acidic (PA), hemagglutinin (HA), nucleoprotein (NP), neuraminidase (NA), matrix protein (MP) and non-structural (NS)] of the new isolated H7N9 virus were amplified using the SuperScript^®^ III One-Step RT-PCR System with Platinum^®^ Taq RT-PCR (Invitrogen Life Technologies, Carlsbad, CA, USA). The reaction volume was 25 μl, including 12.5 μl 2X reaction mix, 1.0 μl SuperScript™ III RT/Platinum® Taq mix, 0.25 μl forward primer (20 μM), 0.25 μl reverse primer (20 μM), 6.0 μl RNase-free water and 5.0 μl template RNA sample. In total, 56 primers were used in the present study and are shown in Table I. The PCR products were sequenced by Takara Bio, Inc. (Dalian, China).

### Phylogenetic analysis

The sequence data were edited and aligned using BioEdit software (https://www.bioedit.com/). The full-genome sequences of the virus isolated in the present study are available from GenBank. The accession numbers of the sequences are: HA, KF420297; NA, KF420299; MP, KF420301; NP, KF420303; NS, KF420305; PA, KF420307; PB1, KF420309; and PB2, KF420311. Online blast analysis demonstrated that all eight gene sequences of A/Changsha/2/2013 (H7N9) were highly homologous to those in the new H7N9 virus strains isolated in mainland China and Taiwan in March-April 2013. The homology was >99%, which indicated that the new isolated virus was the H7N9 virus. The new H7N9 virus gene sequences reported in the influenza virus database between March 1 and August 1, 2013, were retrieved for the construction of phylogenetic trees (7). Phylogenetic trees were constructed using the neighbor-joining method with MEGA software, version 5.2 (http://www.megasoftware.net/megamac.php). The molecular characteristics of each gene were analyzed.

## Results

### Phylogenetic analysis

**Figure 1:**
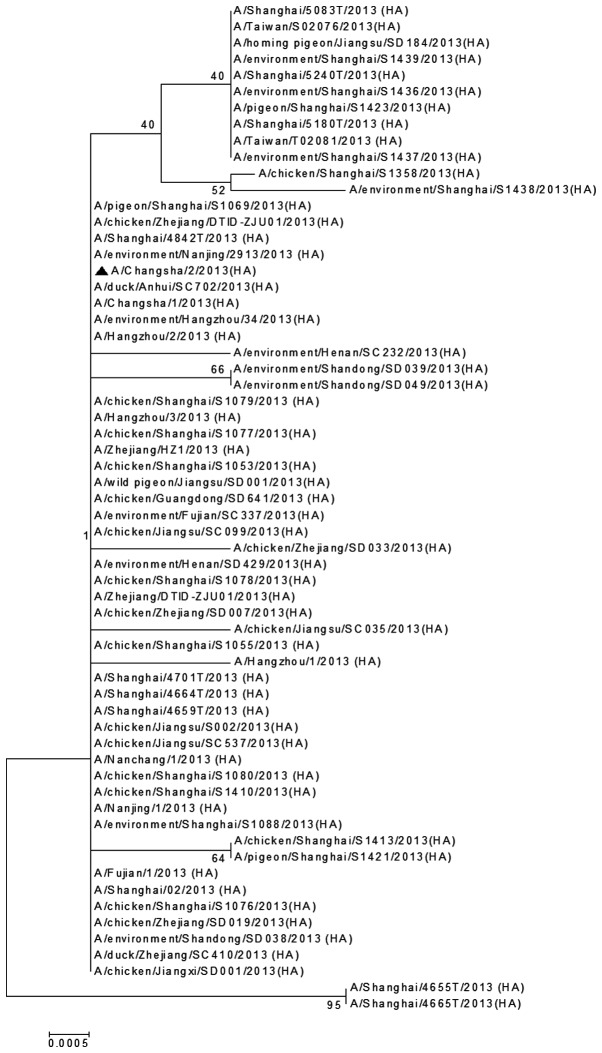
Phylogenetic tree for the hemagglutinin gene of the H7N9 virus isolated from a case in Changsha, China. ▲, sequence of the H7N9 virus in the present study.

The H7N9 virus nucleic acid test of the tracheal aspirate samples from the male patient was positive and the virus was identified as the H7N9 virus [A/Changsha/2/2013 (H7N9)]. The HA gene phylogenetic tree (Fig. 1) showed that the new H7N9 viruses isolated in 2013 were closely genetically associated. All the viruses were in a large cluster, with the exception of two Shanghai strains of the human H7N9 virus (A/Shanghai/4655T/2013 and A/Shanghai/4665T/2013), which formed a sub-branch. The A/Changsha/2/2013 virus in the present study was closely associated with H7N9 viruses of different regions and sources, including H7N9 viruses of human (A/Shanghai/4842T/2013), avian (A/duck/Anhui/SC702/2013) and environmental (A/environment/Nanjing/2913/2013) origin.

**Figure 2:**
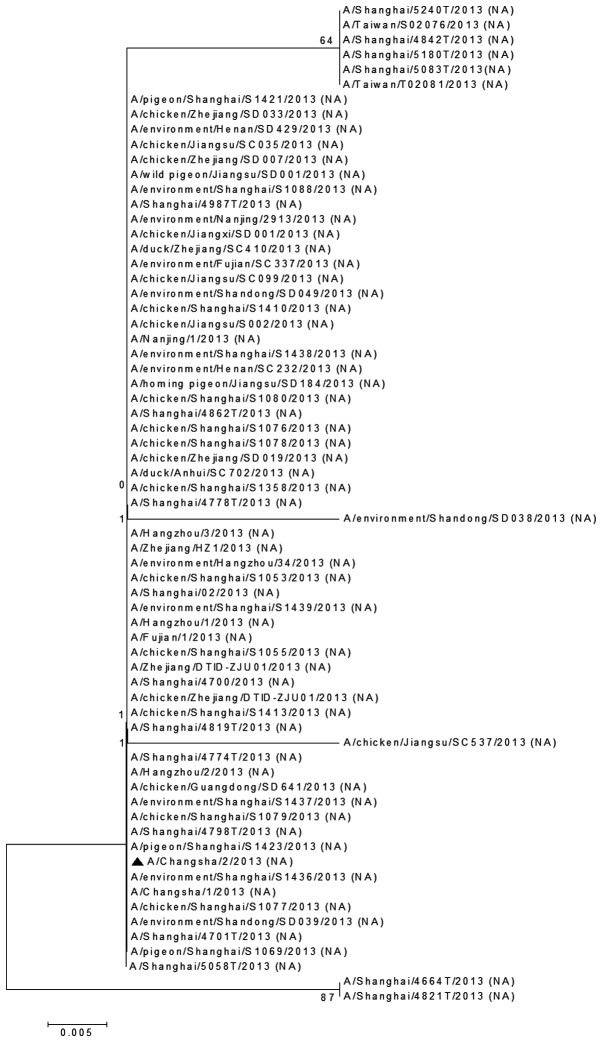
Phylogenetic tree for the neuraminidase gene of the H7N9 virus isolated from a case in Changsha, China. ▲, sequence of the H7N9 virus in the present study.

The NA gene phylogenetic tree (Fig. 2) was similar to that of the HA gene. The sequences of the new H7N9 viruses were also in a large cluster, while two Shanghai strains of the human H7N9 virus (A/Shanghai/4664T/2013 and A/Shanghai/4821T/2013) formed a sub-branch. The A/Changsha/2/2013 H7N9 virus was clustered together with an environmental H7N9 virus (A/environment/Shanghai/S1436/2013), pigeon H7N9 virus (A/pigeon/Shanghai/S1423/2013) and human H7N9 virus (A/Shanghai/4798T/2013) isolated in Shanghai, China.

**Figure 3:**
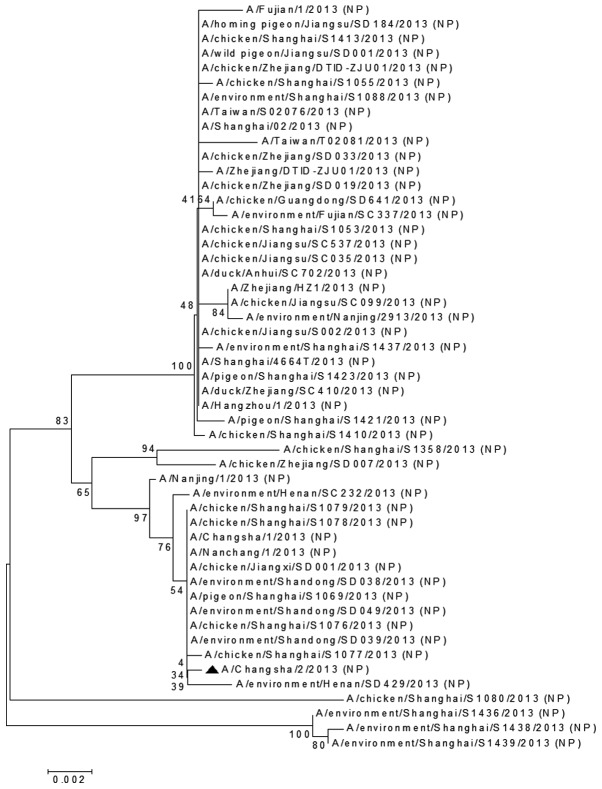
Phylogenetic tree for the nucleoprotein gene of the H7N9 virus isolated from a case in Changsha, China. ▲, sequence of the H7N9 virus in the present study.

**Figure 4:**
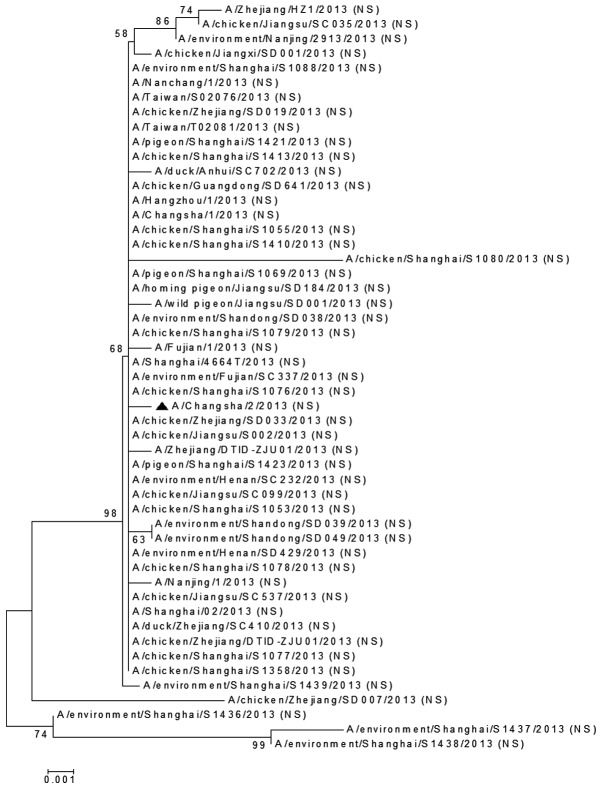
Phylogenetic tree for the non-structural gene of the H7N9 virus isolated from a case in Changsha, China. ▲, sequence of the H7N9 virus in the present study.

The NP (Fig. 3) and NS genes (Fig. 4) of the H7N9 viruses isolated in 2013 formed a phylogenetic tree with two main branches. One branch contained the avian, human and environmental H7N9 viruses isolated from different regions. Another branch contained Shanghai strains of environmental H7N9 viruses. The NP gene of A/Changsha/2/2013 was clustered together on the phylogenic tree with those from the chicken H7N9 virus isolated in Shanghai (A/chicken/Shanghai/S1077/2013), the environmental H7N9 virus strain isolated in Henan, China (A/environment/Henan/SD429/2013) and other H7N9 viruses (Fig. 3). The NS gene of the isolated H7N9 virus from the present case was clustered together with the chicken H7N9 virus isolated in Shanghai (A/chicken/Shanghai/S1076/2013) and the chicken H7N9 virus isolated in Zhejiang, China (A/chicken/Zhejiang/SD033/2013; Fig. 4).

**Figure 5:**
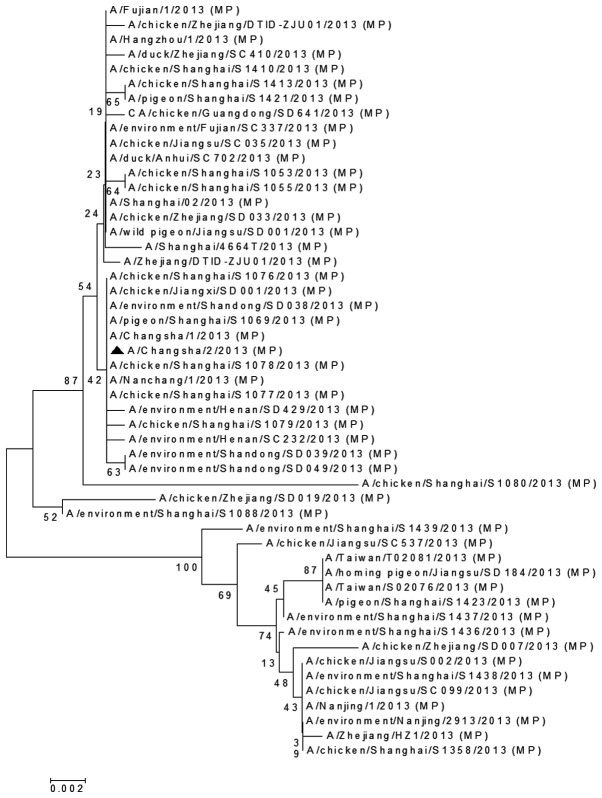
Phylogenetic tree for the matrix protein gene of the H7N9 virus isolated from a case in Changsha, China. ▲, sequence of the H7N9 virus in the present study.

**Figure 6:**
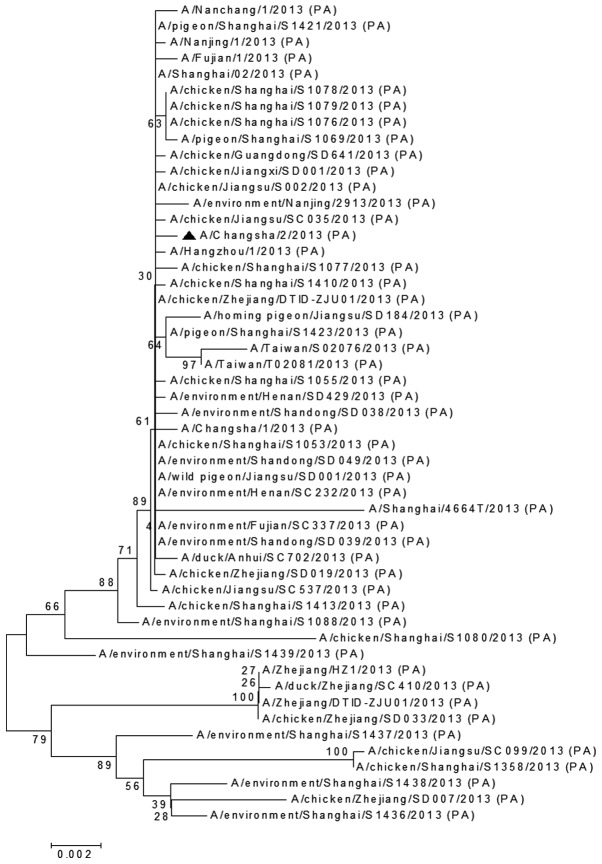
Phylogenetic tree for the polymerase acidic gene of the H7N9 virus isolated from a case in Changsha, China. ▲, sequence of the H7N9 virus in the present study.

**Figure 7:**
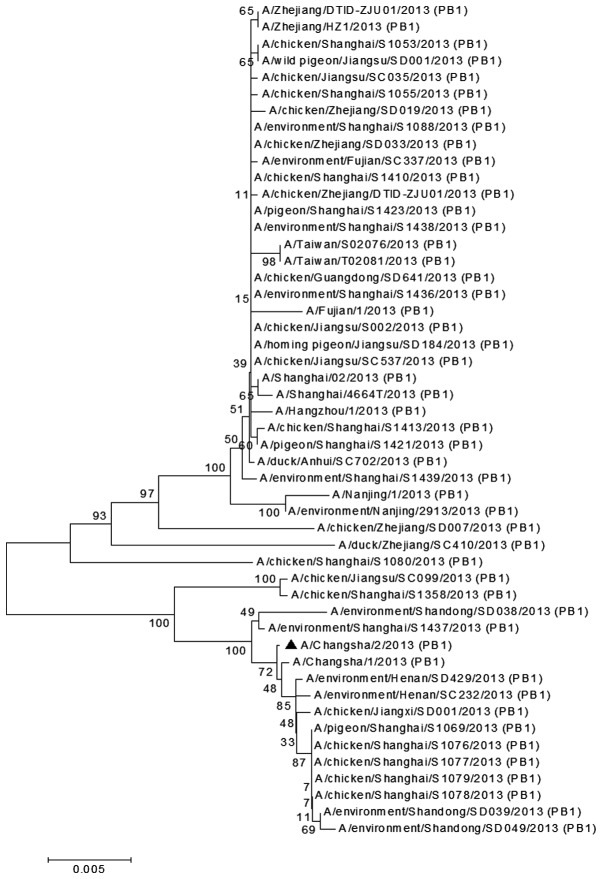
Phylogenetic tree for the polymerase basic protein 1 gene of the H7N9 virus isolated from a case in Changsha, China. ▲, sequence of the H7N9 virus in the present study.

**Figure 8:**
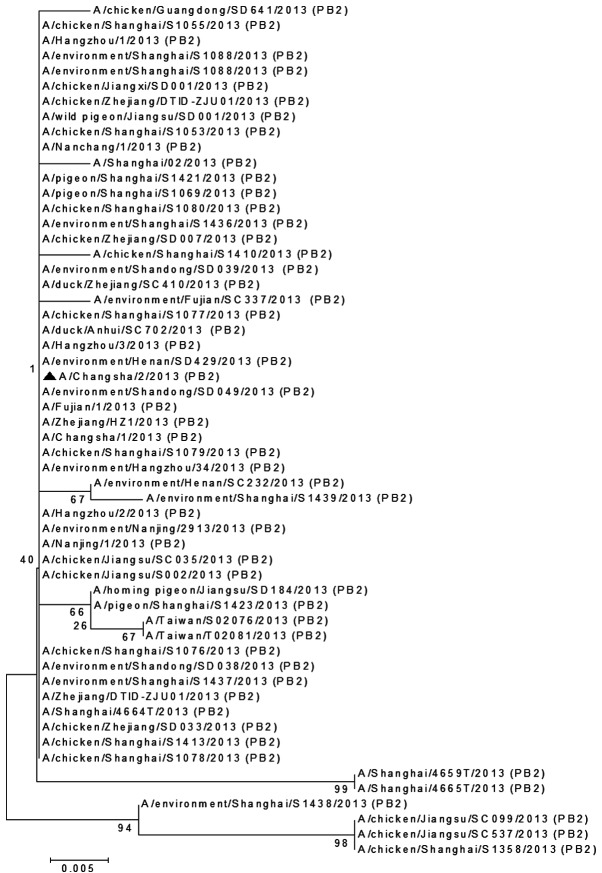
Phylogenetic tree for the polymerase basic protein 2 gene of the H7N9 virus isolated from a case in Changsha, China. ▲, sequence of the H7N9 virus in the present study.

The MP (Fig. 5), PA (Fig. 6), PB1 (Fig. 7) and PB2 (Fig. 8) genes of the H7N9 viruses isolated in 2013 also formed phylogenetic trees with two main branches; however, each branch contained avian, human and environmental H7N9 viruses of different regions. The phylogenetic tree demonstrated that the MP gene of A/Changsha/2/2013 was closely associated with the avian H7N9 virus isolated in Shanghai (A/chicken/Shanghai/S1078/2013) and the human H7N9 virus isolated in Nanchang, China (A/Nanchang/1/2013; Fig. 5). The PA gene of A/Changsha/2/2013 was clustered together on the phylogenic tree with the chicken H7N9 virus (A/chicken/Jiangsu/SC035/2013), chicken H7N9 virus (A/chicken/Shanghai/S1077/2013) and human H7N9 virus (A/Hangzhou/1/2013; Fig. 6). The phylogenetic tree of the PB1 gene demonstrated that the A/Changsha/2/2013 H7N9 virus was closely associated with certain environmental H7N9 viruses (A/environment/Henan/SD429/2013 and A/environment/Henan/SC232/2013; Fig. 7). The PB2 gene of A/Changsha/2/2013 was clustered together on the phylogenic tree with environmental H7N9 viruses isolated in Henan (A/environment/Henan/SD429/2013) and Shandong, China (A/environment/Shandong/SD049/2013; Fig. 8). These results suggest that the A/Changsha/2/2013 strain is evolutionally close to other H7N9 avian influenza virus stains reported by Genbank between March and August 2013.

### Characteristics of the viral genes

In order to analyze the key mutation sites, the genome sequence of A/Changsha/2/2013 (H7N9) was compared with those of the new H7N9 viruses reported in the influenza virus database in 2013. There was only one single amino acid arginine (R) at the protein cleavage site (amino acids 339–345) of the linker peptide between HA1 and HA2 of the A/Changsha/1/2013 (H7N9) HA gene, which was similar to those in other newly isolated H7N9 viruses, indicating that the virus had a low pathogenicity for poultry (5). The Q226L amino acid mutation (the amino acid site encoded by the H3 type influenza virus corresponded to amino acid 235 in the present study) occurred in the HA protein of the new H7N9 viruses of human, avian and environmental origin. The mutation rate was 88.85% (46/52). G228S and G186V mutations were also revealed to be present at the receptor binding site, with a mutation rate of 100%. However, no A138S mutation was identified (Table II). The PB2 gene of eight viruses among 51 new H7N9 viruses harbored the E627K mutation. However, only one virus harbored the D701N mutation. No E627K and D701N mutations were detected in the A/Changsha/2/2013 PB2 protein (Table II). Amino acid deletions were also identified in the NA coding protein at amino acids 69–73, and the NS1-coding protein at amino acids 218–230 of all the new H7N9 viruses that were isolated in 2013, respectively. Resistance gene loci analysis showed that the R294K mutation did not occur in the NA coding protein of the A/Changsha/2/2013 virus and other 50 H7N9 virus strains. However, it occurred in a human H7N9 virus strain isolated in Taiwan. The S31N mutation of the M2 protein encoded by the MP gene was identified in the 52 new H7N9 virus strains. Other mutations in the new H7N9 virus isolated in 2013 included: P42S in the NS1 protein encoded by the NS gene; N30D and T215A in the M1 protein encoded by MP gene; and L89V in the PB2 protein (Table II). These results suggest that the A/Changsha/2/2013 H7N9 virus strain has molecular characteristics that may facilitate adaptation of the virus to mammalian hosts and may even bind to human receptors.

## Discussion

The phylogenetic tree results demonstrated that the HA and NA genes of the A/Changsha/2/2013 (H7N9) virus were closely associated with those of the other new H7N9 viruses isolated in 2013. All the viruses were in a large cluster, while the two Shanghai strains of the human H7N9 virus (A/Shanghai/4664T/2013 and A/Shanghai/4821T/2013) formed a sub-branch. The results indicate that the new human H7N9 viruses were highly homologous to avian and environmental H7N9 viruses. It further confirmed that the H7N9 virus was transformed from poultry to humans. More H7N9 viruses were isolated in different birds and environments, suggesting that H7N9 viruses have been distributed in these avian species and environments. The NP and NS genes of the H7N9 viruses isolated in 2013 formed a phylogenetic tree with two main branches. One branch contained avian, human and environmental H7N9 viruses isolated in different regions. Another branch contained three environmental H7N9 viruses isolated in Shanghai. The MP, PA, PB1 and PB2 genes of the H7N9 viruses formed phylogenetic trees with two main branches and each branch contained avian, human and environmental H7N9 viruses isolated in different regions. This suggests that NP, NS, MP, PA, PB1 and PB2 genes of the new H7N9 viruses isolated in 2013 are in different degrees of evolutionary separation. The different branches indicate the genetic diversity of the H9N2 viruses from which the genes originate (8).

The HAs of human influenza viruses bind to cell sialic acid linked to galactose by an α-2,6 linkage, as found on human cells, while avian viruses have a predilection for sialic acid linked to galactose by α-2,3 linkages, as found on avian epithelia. This receptor specificity is considered to be one of the factors responsible for the species barrier that prevents avian viruses from readily infecting humans. Avian influenza viruses typically contain Gln226 in the HA gene encoding protein, while human viruses contain Leu226 (8–11). The HA protein of A/Changsha/2/2013 and other 45 other new human, avian and environmental H7N9 viruses harbored the Q226L mutation (with a mutation rate of 88.5%) in the receptor binding site. In addition, 52 H7N9 viruses harbored the G186V and G228S mutations in the receptor binding site of the HA protein. This indicates that A/Changsha/2/2013 and other new H7N9 viruses specifically bind to human receptors and are able to infect humans (8–10,12).

The E627K mutation of the PB2 polymerase protein was identified in H5N1 and H7N7 avian influenza viruses isolated from several cases of mortality. The E627K and D701N mutations favor the efficient replication and dissemination of avian influenza viruses in mammals (2,13–15). Although no E627K and D701N mutations were detected in the PB2 protein of A/Changsha/2/2013, the E627K mutation and D701N mutation occurred in eight human H7N9 virus strains and one human H7N9 virus strain among the 51 new H7N9 viruses, respectively. However avian and environmental H7N9 viruses had no E627K and D701N mutations. This demonstrates that the new H7N9 viruses isolated in 2013 enhanced the adaptive capacity in the mammalian host. However, further investigation is required.

A deletion at amino acids 69–73 in the NA protein has been reported to increase viral virulence in mammals (11), while a deletion at amino acids 218–230 in the NS1 protein has been reported to reduce viral virulence in mammals (16). The NA and NS1 proteins of all the new H7N9 viruses isolated in 2013, including A/Changsha/1/2013, have deletions at amino acids 69–73 and 218–230, respectively. Resistance gene loci analysis demonstrated that the R294K mutations of the NA coding protein only occurred in one H7N9 virus strain isolated in Taiwan (A/Taiwan/S02076/2013), suggesting that A/Changsha/2/2013 and 50 other new H7N9 viruses isolated in 2013 were sensitive to neuraminidase inhibitors (e.g. oseltamivir) (17). As neuraminidase inhibitors are widely used in the treatment of H7N9 virus infection, the possible development of drug resistance requires close monitoring. All 52 H7N9 viruses were resistant to the M2 ion channel blocker amantadine. A/Changsha/2/2013 (H7N9) was isolated from a severe, fatal case of pneumonia.

The main genetic characteristic of the A/Changsha/2/2013 (H7N9) virus is that it is able to infect humans. The new H7N9 viruses are distributed in different avian species and environments and are capable of infecting humans. This suggests that the next phase of H7N9 avian influenza prevention and control is urgently required.

## Figures and Tables

**Table I:** Primers used in the study.

Primer	Sequence (5′-3′)	Product (bp)
HA-1-F	ATGAACACTCAAATCCTGGT	978
HA-1-R	TGCTAGCAGCAGACTCCTTT	
HA-2-F	ACCAAGCTATATGGGAGTGG	832
HA-2-R	CAAAGCAACCAGTGCCATCT	
HA-3-F	TTGCCATTTCAGAACATAGA	810
HA-3-R	GTAGAAACAAGGGTGTTTTT	
NA-1-F	ATGAATCCAAATCAGAAGAT	835
NA-1-R	CGTAACATGAGCATTCTTCA	
NA-2-F	GTAGTATGGTACAACAGAAGG	623
NA-2-R	TAGTCCATGAAAGATCCACT	
NA-3-F	CAGATAGACCCAGTAGCAAT	486
NA-3-R	CTCTATTTTAGCCCCATCAG	
NA-4-F	ATGACCCTTATCCAGGTAAT	426
NA-4-R	AGAATAAACAAGGGTCTTTT	
MP-1-F	ATGAGTCTTCTAACCGAGGT	767
MP-1-R	CTAGAGGCTCACTTGAACCG	
MP-2-F	TACTACTAACCCACTAATTAGG	502
MP-2-R	AGTAGAAACAAGGTAGTTTT	
NP-1-F	ATGGCGTCTCAAGGCACCAA	790
NP-1-R	GTGCAGACCTTGCCAGAAAA	
NP-2-F	GCATATGAGAGAATGTGCAA	818
NP-2-R	CCGAAGAAATAAGATCCTTCAT	
NP-3-F	ACTCTGCAGCGTTTGAGGAC	520
NP-3-R	AGTAGAAACAAGGGTATTTT	
NS-1-F	ATGGATTCCAATACTGTGTC	652
NS-1-R	ACTTTGTAGAGAGTGGAGATC	
NS-2-F	TCAGTGTGATTTTCAATCGG	464
NS-2-R	AGTAGAAACAAGGGTGTTTT	
NS-3-F	TCAGTGTGATTTTCAATCGG	355
NS-3-R	CAAAGCTATTCTCCGTAATT	
PA-1-F	ATGGAAGACTTTGTGCGACA	1000
PA-1-R	AATTGGGGTTTATGCCTTTC	
PA-2-F	CTCCCTGCTCTCAGCGGTCGAAAT	782
PA-2-R	TGGTTCCAACCTTGGGTCGG	
PA-3-F	GCATCTTGTGCAGCCATGGA	606
PA-3-R	CCTAAGCGCCTGAACAATGA	
PA-4-F	CAATGGGACCTCCAAGATCA	381
PA-4-R	AGGCACTCCTCGATTGCTTC	
PA-5-F	CAATGGGACCTCCAAGATCA	509
PA-5-R	AGTAGAAACAAGGTACTTTT	
PB1-1-F	ATGGATGTCAATCCGACTTT	962
PB1-1-R	ATTGCTAGAAACATCCGGGG	
PB1-2-F	GCAAGCTGAAAAGGAGGGCA	1000
PB1-2-R	TGCGTATCACCCCTGTGACA	
PB1-3-F	TTCGTAGCTAACTTCAGTATG	817
PB1-3-R	AGTAGAAACAAGGCATTTTT	
PB1-4-F	TTCGTAGCTAACTTCAGTATG	800
PB1-4-R	TTTTCATGAAGGACAAGCTA	
PB2-1-F	ATGGAAAGAATAAAAGAACTAAG	992
PB2-1-R	TTGAAAGTGAAACCTCCAAA	
PB2-2-F	TAGAAGAGCAACAGTATCAGC	1000
PB2-2-R	GAATAGAACCCTCACGAACC	
PB2-3-F	CCATGATGTGGGAGATCAAT	703
PB2-3-R	GGTCGTTTTTAAACAATTCG	
PB2-4-F	CCATGATGTGGGAGATCAAT	714
PB2-4-R	AGTAGAAACAAGGTCGTTTT	

PB2, polymerase basic protein 2; PB1, polymerase basic protein 1; PA, polymerase acidic; HA, hemagglutinin; NP, nucleoprotein; NA, neuraminidase, MP, matrix protein; NS, non-structural.

**Table II:** Molecular analysis of important amino acids in the HA, NA, NS1, M1, M2, PB1 and PB2 proteins of the H7N9 virus.

Gene	Site	Position	A/Changsha/2/2013	Mutated virus	Non-mutated virus
HA	Q226L	226/235^a^	L	46/52	6/52
	G228S	228/237^a^	G	52/52	0/52
	A138S	138/130^a^	A	0/52	52/52
	G186V	186/195^a^	V	52/52	0/52
NA	R294K	294/289^b^	R	1/52	51/52
		69–73	Deletion	52/52 deletion	0/52 no deletion
NS1	P42S	42	S	50/50	0/50
		218–230	Deletion	50/50 deletion	0/50 no deletion
M1	N30D	30	D	50/50	0/50
	T215A	215	A	50/50	0/50
M2	S31N	31	N	50/50	0/50
PB1	H99Y	99	H	0/48	48/48
PB2	L89V	89	V	51/51	0/51
	E627K	627	E	8/51	43/51
	D701N	701	D	1/51	50/51

a , amino acid sites of H3/H7N9;

b , amino acid sites of avian N9/H7N9.

PB2, polymerase basic protein 2; PB1, polymerase basic protein 1; PA, polymerase acidic; HA, hemagglutinin; NP, nucleoprotein; NA, neuraminidase, M, matrix protein; NS, non-structural.
